# Prediction Model for Quality Changes in Repeatedly Frozen–Thawed Pork Based on MRI Scans and Chemometrics

**DOI:** 10.3390/foods15040686

**Published:** 2026-02-13

**Authors:** Hui Liu, Yuhui Zhang, Ke Liu, Wusun Li, Xiaoyan Tang

**Affiliations:** Key Laboratory of Agro-Product Quality and Safety, Institute of Quality Standard & Testing Technology for Agro-Products, Chinese Academy of Agricultural Sciences, Beijing 100081, China

**Keywords:** pork quality, water-holding capacity (WHC), freeze–thaw cycles, magnetic resonance imaging (MRI), chemometric modeling

## Abstract

This study investigated fresh pork and pork subjected to repeated freeze–thaw cycles. The effects of freeze–thaw treatments on water status, WHC, and quality attributes of pork were systematically analyzed, and a nondestructive prediction method for WHC based on magnetic resonance imaging (MRI) was developed. The results showed that increasing freeze–thaw cycles significantly reduced moisture content and increased drip loss, indicating a continuous deterioration of overall WHC. Texture parameters and shear force values decreased markedly, suggesting that muscle structure was progressively damaged by ice crystal formation and recrystallization. T_2_-weighted MRI pseudo-color scans clearly reflected changes in internal water distribution, with high-signal regions gradually decreasing as freeze–thaw cycles increased, which was consistent with the experimentally measured trends in moisture content and WHC. Based on MRI features, principal component regression (PCR) and partial least squares regression (PLSR) models were established to predict pork WHC. The PCR model extracted 16 principal components (cumulative contribution rate of 96.394%), with calibration set results of Rc^2^ = 0.8825 and RMSEC = 1.7959, and validation set results of Rp^2^ = 0.8856 and RMSEP = 2.0284. The optimal number of latent variables for the PLSR model was six, yielding calibration set results of Rc^2^ = 0.9634 and RMSEC = 1.0026, and validation set results of Rp^2^ = 0.9656 and RMSEP = 1.1119, with all residuals less than 1. Overall, the combination of MRI and chemometric methods, particularly the PLSR model, enables rapid, nondestructive, and accurate prediction of pork WHC, providing a useful tool for quality evaluation under repeated freeze–thaw conditions and for quality control in pork processing, storage, and cold-chain management.

## 1. Introduction

Pork is one of the most important meat products in the human diet, and the evaluation and control of its quality attributes have long been a major focus in meat science research [1]. Among these attributes, water-holding capacity (WHC) is a key indicator of pork quality, as it directly affects tenderness, juiciness, nutritional value, and economic value [2]. However, traditional methods for WHC evaluation are often labor-intensive and time-consuming, making them unsuitable for rapid and online quality detection in modern meat processing and quality control systems [3]. Poor WHC leads to excessive fluid loss during storage and processing, resulting in reduced consumer acceptance and economic losses for the meat industry. Various methods have been developed to evaluate WHC, including the gravimetric bag method, filter paper press method, centrifugation-based techniques, and low-field nuclear magnetic resonance (LF-NMR) relaxation measurements. Conventional methods are simple and widely used but are often destructive and highly dependent on experimental conditions. In contrast, LF-NMR provides a rapid and nondestructive approach for characterizing water mobility and distribution in muscle foods [3]. In the assessment of meat WHC, investigating the relationship between water status and distribution in pork with different WHC levels is of great importance. Furthermore, the development of WHC evaluation methods based on machine vision techniques has significant practical potential.

In China, the cold-chain logistics system is still not fully developed. During the stages of slaughtering, initial chilling, cold storage, transportation, and thawing of frozen pork, temperature fluctuations and repeated freeze–thaw cycles are common. Previous studies have shown that repeated freeze–thaw cycles cause mechanical damage to muscle cell membranes due to ice crystal formation and recrystallization, while also accelerating protein denaturation and lipid oxidation [4]. These changes weaken the ability of muscle tissue to retain water, ultimately leading to a significant reduction in WHC [4,5]. Although conventional WHC measurement methods, such as pressure loss, drip loss, and cooking loss, can provide quantitative evaluation, they have clear limitations. The pressure loss method requires the application of external force and a resting time of more than 30 min, while the drip loss method may take up to 24 h. In addition, these methods cause irreversible damage to the samples and cannot be used for dynamic monitoring of meat quality [6]. Therefore, understanding the mechanisms by which freeze–thaw cycles affect water status, WHC, and quality loss in pork is essential for effective cold-chain quality control. The development of a nondestructive, rapid, and accurate method for WHC determination has become an important research goal in meat science.

Magnetic resonance imaging (MRI) is based on the principle of nuclear magnetic resonance and can visualize the internal water status and spatial distribution of samples by detecting hydrogen proton signals without damaging the samples [7,8]. Due to its high sensitivity, visualization capability, non-invasiveness, and relative cost-effectiveness in terms of operational efficiency, MRI has shown increasing application potential in food science, particularly in meat quality evaluation [9]. Tan et al. [10] used MRI to monitor water distribution changes during the drying of mushrooms and oysters and found that changes in MRI signal intensity were highly consistent with the decreasing trend of moisture content [11]. Caballero et al. [12] combined MRI with fractal analysis to predict moisture and fat content in pork, achieving correlation coefficients of 0.683–0.830 for fresh samples. However, studies focusing on quantitative prediction of pork WHC based on MRI are still limited. Most existing work focuses on two-dimensional data, with insufficient exploration of the quantitative relationship between image features and WHCs and a lack of systematic analysis of how repeated freeze–thaw cycles affect the relationship between MRI signals and WHCs [2,8].

In this study, fresh pork and pork subjected to repeated freeze–thaw treatments were used as experimental samples to simulate quality changes caused by temperature fluctuations during real cold-chain circulation. T_2_-weighted MRI scans were acquired, and key image features, including signal intensity, regional distribution, and texture characteristics, were extracted. Principal component regression (PCR) and partial least squares regression (PLSR) models were then established to predict pork WHC based on MRI features. The performance of the two models was further compared, aiming to provide scientific evidence and technical support for rapid detection, online monitoring of pork WHC, and guidance for pork processing, storage, and cold-chain logistics management.

## 2. Materials and Methods

### 2.1. Sample Collection and Treatment

To ensure internal consistency, six pigs reared under identical conditions were selected from Beijing Ershang Meat Food Group Co., Ltd. (Beijing, China). The longissimus dorsi muscles from the left side of each carcass were collected (approximately 2 kg per sample). After removing visible fat and connective tissue, each sample was cut perpendicular to the muscle fiber direction into four equal portions (about 500 g each). One portion was used without freeze–thaw treatment and designated as 0 F–T, while the remaining three portions were subjected to 3, 5, and 10 freeze–thaw cycles, respectively, and labeled as 3 F–T, 5 F–T, and 10 F–T. Each freeze–thaw cycle consisted of freezing at −18 °C for 12 h followed by complete thawing at 4 °C. All measurements were conducted after the designated treatments.

### 2.2. Determination of Moisture Content

Moisture content of fresh pork and pork subjected to different freeze–thaw cycles was determined according to the Chinese National Standard GB 5009.3-2016 using the direct drying method [13]. Experimental procedures were performed following the standard protocol. Moisture content was calculated as follows: W = [(m_3_ − m_1_)/(m_2_ − m_1_] × 100, where W is the moisture content (%); m_1_ is the mass of the weighing bottle (g); m_2_ is the mass of the weighing bottle with sample (g); and m_3_ is the mass of the weighing bottle with dried sample (g).

### 2.3. Determination of Water-Holding Capacity

#### 2.3.1. Drip Loss

Drip loss was measured according to the method of Honikel (1998) [14]. Pork samples were cut along the muscle fiber direction into strips of 2 cm × 3 cm × 5 cm, accurately weighed (±0.01 g), and suspended in sealed bags at 4 °C for 24 h. Afterwards, surface moisture was gently removed and samples were reweighed. Drip loss was calculated as follows: DL = [(m_1_ − m_2_)/m_1_] × 100, where DL is drip loss (%); m_1_ is the initial mass (g); and m_2_ is the mass after suspension (g).

#### 2.3.2. Cooking Loss

Cooking loss was determined following the method described by Li et al. (2006) [15]. Pork samples (2.5 cm thickness) were weighed (±0.01 g) and cooked in a water bath at 72 °C until the core temperature reached 70 °C. Samples were then cooled under running water for 30 min, stored at 4 °C for 24 h, blotted dry, and reweighed. Cooking loss was calculated as follows: CL = [(m_1_ − m_2_)/m_1_] × 100, where CL is cooking loss (%); m_1_ is the mass before cooking (g); and m_2_ is the mass after cooking (g).

#### 2.3.3. Pressing Loss

Pressing loss was measured according to the method of Farouk et al. (2004) [16]. Cylindrical samples (2.5 cm diameter, 1.0 cm thickness) were obtained perpendicular to the muscle fiber direction and weighed (±0.01 g). Each sample was wrapped with 16 layers of filter paper on both sides and pressed at a constant load of 35.0 kg for 5 min using a meat press. After removing the filter paper, samples were immediately reweighed. Pressing loss was calculated as follows: PL = [(m_1_ − m_2_)/m_1_] × 100, where PL is pressing loss (%); m_1_ is the mass before pressing (g); and m_2_ is the mass after pressing (g).

### 2.4. Shear Force Measurement

Samples used for shear force measurement were obtained from cooked meat after cooking loss determination. Meat samples were cut along the muscle fiber direction into strips 1.0 cm thick, free of connective tissue and visible fat. Shear force was measured using a meat tenderness analyzer and expressed in newtons (N).

### 2.5. Texture Profile Analysis (TPA)

Fresh pork and freeze–thaw-treated samples were cut along the muscle fiber direction into cubes of approximately 1 cm^3^. Texture profile analysis was performed at room temperature using a TA-XT2i texture analyzer equipped with a P36/R probe. Two compression cycles were applied with a compression ratio of 50% and a 5 s interval between cycles. Pre-test, test, and post-test speeds were set at 2 mm/s, 1 mm/s, and 5 mm/s, respectively [17]. Texture parameters including hardness, cohesiveness, springiness, chewiness, and resilience were recorded [18].

### 2.6. Acquisition of T_2_-Weighted MRI Scans

T_2_-weighted MRI scans were acquired using a multi-slice spin echo (SE) sequence, modified from the method of Cheng et al. [10]. Imaging parameters were as follows: 4 slices; field of view (FOV) 100 mm × 100 mm; slice thickness 4 mm; slice gap 0.5 mm; matrix size 256 (readout) × 192 (phase); number of averages 4; repetition time (TR) 1600 ms; and echo time (TE) 50 ms. Images were saved in DICOM format for subsequent analysis.

### 2.7. Extraction of DICOM Image Features

Due to differences in the placement position and size of the samples in NMR tubes, some slice images cannot fully display the pork contours (such as missing edges or local blurring). For each sample, one slice with a clear image and complete contour is selected for subsequent analysis to ensure the accuracy of feature extraction.

Using the dicomread function, DICOM files can be read in the MATLAB environment to obtain a 256 × 256 two-dimensional matrix. The values in the matrix represent the grayscale values of the image.

The key code for reading and displaying DICOM images using the dicomread function in MATLAB is as follows:

*% Read the image*

image = dicomread(name);

*% Convert the image to grayscale*

grayImage = image;

*% Convert each pixel value in grayImage from its original data type to double type, and store these converted double-precision values in the variable data*

data = double(grayImage);

*% Write the data from the matrix to a text file*

Dlmwrite (‘grayImageData.txt’, data, ‘delimiter’, ‘\t’).

The matrix corresponding to each image is associated one-to-one with the pressure loss and transformed into a 256 × 256 × *n* three-dimensional matrix.

### 2.8. Regression Model Construction and Evaluation

Principal component regression (PCR) and partial least squares regression (PLSR) models were developed using MATLAB R2022a to predict pork WHC. For the PLSR model, Monte Carlo cross-validation (MCCV) was applied to validate model performance. Model accuracy and reliability were evaluated using the coefficient of determination for the prediction set (Rp^2^), root mean square error of prediction (RMSEP), and mean squared error of prediction (MSEP).

(a) **Coefficient of determination**

The coefficient of determination includes the coefficients of determination for the calibration set ($R_{c}^{2}$) and the prediction set ($R_{p}^{2}$), which are used to evaluate the goodness of fit and predictive performance of the developed models. The values range from 0 to 1, and values closer to 1 indicate better model fitting and stronger explanatory ability. The coefficient of determination is calculated as follows:

$$R_{c}^{2}{,R}_{p}^{2}=1-\frac{\Sigma_{i=1}^{N}{\left(y_{act}-y_{pre}\right)}^{2}}{\sum_{i=1}^{N}{\left(y_{act}-\bar{y}\right)}^{2}}$$

(b) **Root mean square error (RMSE)**

The root mean square error includes the RMSE of calibration (RMSEC) and the RMSE of prediction (RMSEP), which are used to assess the accuracy and robustness of the constructed models. Lower RMSE values indicate smaller prediction errors and higher model accuracy. The RMSE is calculated as follows:

$$RMSEC,RMSEP=\sqrt{\frac{1}{N}\sum_{i=1}^{N}{\left(y_{act}-y_{pre}\right)}^{2}}$$

(c) **Residual**

The residual (Res) represents the difference between the actual observed value and the predicted value and is commonly used to evaluate model fitting performance. Smaller residuals indicate that the predicted values are closer to the actual values, suggesting a better fit of the model. Conversely, larger residuals imply that the model may not adequately capture the underlying relationships in the data and may require further optimization. The residual is calculated as follows:

$$\mathrm{Res}=y_{act}-y_{pre}$$

where N denotes the number of samples, $y_{\mathrm{act}}$ and $y_{\mathrm{pre}}$ represent the actual and predicted values, respectively, and $\bar{y}$ is the mean value of all samples in the calibration or prediction dataset.

### 2.9. Statistical Analysis

Each experimental group was repeated at least three times; each repetition included six sample replicates, and each sample was measured in triplicate. Statistical analysis was performed using SPSS Statistics 26.0. One-way analysis of variance (ANOVA), Duncan’s multiple range test, and Pearson’s correlation analysis were applied, with significance set at *p* < 0.05. Results are expressed as mean ± standard deviation. Graphs were generated using Origin 8.0.

## 3. Results and Discussion

### 3.1. Changes in Moisture Content of Pork

**Figure 1:**
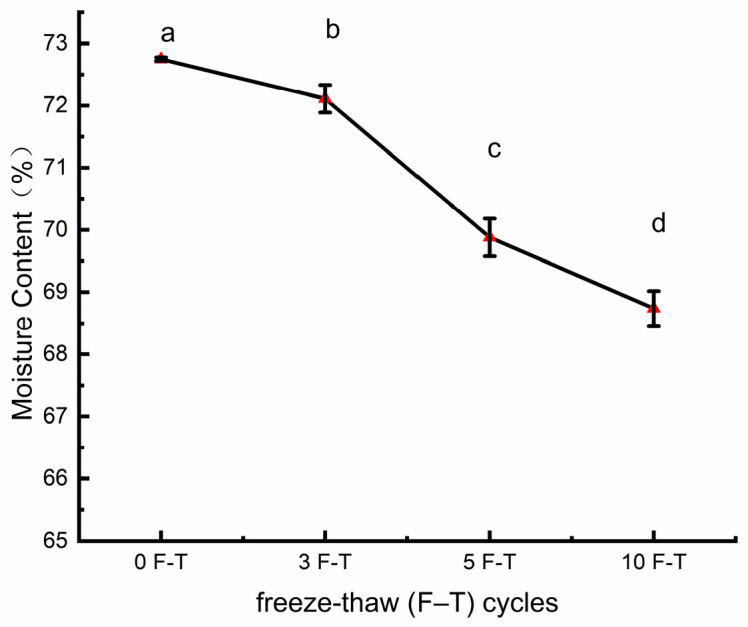
Changes in moisture content of pork. Note: Different letters (a–d) indicate significant differences (*p* < 0.05).

Moisture is the most abundant component in pork and plays a decisive role in sensory qualities such as juiciness and tenderness. As shown in Figure 1, the percentage moisture content of pork significantly decreased with increasing freeze–thaw (F–T) cycles (*p* < 0.05 compared with 0 F–T). This trend indicates that with each cycle of ice crystal formation and melting, water within the muscle tissue is gradually released. Previous studies have reported that repeated freeze–thaw cycles significantly increase thawing loss and decrease the wet weight of meat [19]. In addition, multiple F–T cycles can induce structural disruption and denaturation of myofibrillar proteins, reducing their water-binding capacity [20]. Therefore, moisture loss is mainly attributed to ice crystal-induced damage of muscle cell structures, particularly the intra-myofibrillar regions and sarcolemma, which promotes the transformation of bound and immobilized water into free water that is lost during thawing. Continuous moisture loss subsequently impairs meat quality by decreasing palatability and tenderness, reducing nutritional value and yield, and accelerating quality deterioration through microstructural damage and protein oxidation [11].

### 3.2. Changes in Pork Water-Holding Capacity

Water-holding capacity is a critical indicator for evaluating meat quality, affecting both sensory attributes and economic value [21]. To further assess the effect of repeated freeze–thaw cycles on pork WHC, drip loss, cooking loss, and pressing loss were measured.

**Figure 2:**
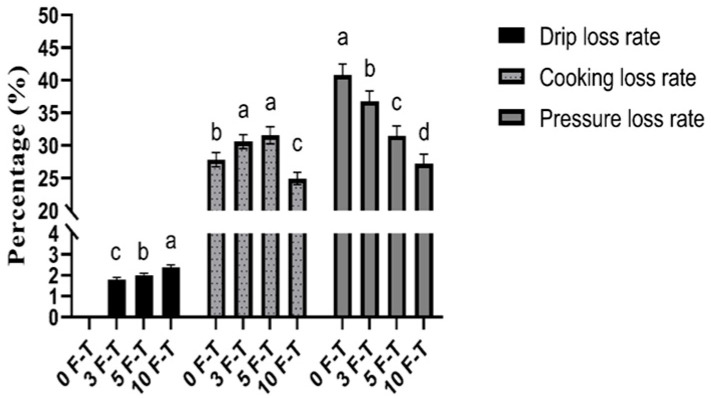
Changes in drip loss, cooking loss and pressure loss of pork. Note: Different letters (a–d) indicate significant differences (*p* < 0.05).

As shown in Figure 2, drip loss significantly increased with repeated F–T cycles (*p* < 0.05), indicating that cell membranes and muscle fibers were repeatedly disrupted during ice crystal formation and melting, causing bound and immobilized water to migrate to free water and be lost during thawing. Previous studies confirm that repeated freeze–thaw cycles enlarge and redistribute ice crystals, damage muscle microstructure, and reduce WHC, leading to increased drip/thawing loss [19]. Cooking loss showed a nonlinear trend, initially increasing and then decreasing. Compared with 0 F–T, 3 F–T and 5 F–T samples showed increased cooking loss, likely due to structural damage and protein denaturation causing free water to be easily expelled. After more cycles (10 F–T), further irreversible protein crosslinking or gelation may stabilize the residual network, partially retaining water or forming a denser fat/protein matrix, resulting in reduced cooking loss. Similar nonlinear trends have been reported for different meat types, muscles, or pre-treatments, suggesting that cooking loss is affected by muscle composition, initial structural damage, and heat-induced protein rearrangement [22]. In contrast, pressing loss decreased significantly with increasing F–T cycles (*p* < 0.05), dropping from 42.23% in 0 F–T to 28.99% after 10 cycles, a reduction of approximately 31%. Although overall WHC decreases with repeated freeze–thaw, different measurement methods (drip, cooking, pressing) have varying sensitivity to water states, and results may not always be consistent depending on muscle type and pre-treatment [23]. Repeated freeze–thaw cycles significantly reduce overall water-holding capacity (WHC) by damaging muscle microstructure, thereby accelerating quality deterioration [24]. To preserve pork quality and economic value, it is recommended to minimize freeze–thaw events to slow the decline in WHC.

### 3.3. Changes in Pork Texture and Shear Force

Texture profile analysis (TPA) quantifies hardness, cohesiveness, springiness, and other texture characteristics in meat and meat products. TPA of fresh and freeze–thaw-treated pork is shown in Table 1. Hardness and gumminess significantly decreased with increasing F–T cycles (*p* < 0.05), indicating that repeated ice crystal formation and rupture loosened the muscle fiber structure [25]. Cohesiveness and resilience did not differ significantly between 3 F–T and fresh meat but decreased significantly in 5 F–T and 10 F–T samples (*p* < 0.05), showing that early freeze–thaw cycles maintain partial structural integrity while cumulative damage later reduces elasticity [26]. Chewiness and gumminess decreased significantly from 3 F–T (*p* < 0.05) and springiness was lower than fresh meat in all F–T groups (*p* < 0.05), indicating that protein denaturation, connective tissue rupture, and water migration jointly weakened structural recovery [27].

**Figure 3:**
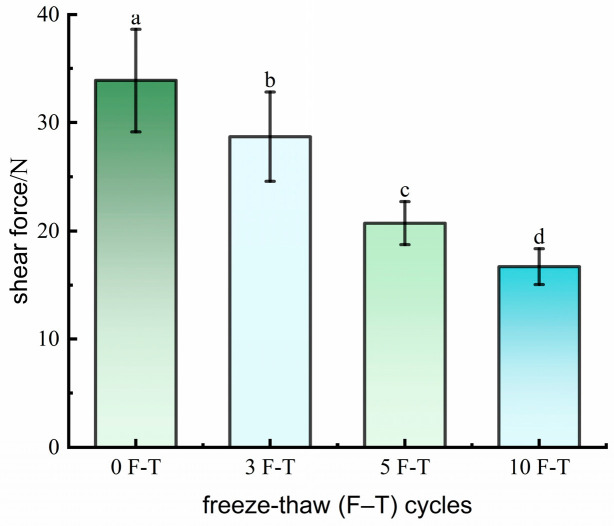
Changes in shear force of pork. Note: Different letters (a–d) indicate significant differences (*p* < 0.05).

Shear force significantly decreased with increasing F–T cycles (*p* < 0.05), dropping by 55.16% after 10 cycles (36.16 N → 15.87 N, Figure 3), indicating a marked reduction in mechanical strength. Repeated freeze–thaw causes surface and internal fiber breakage, increased myofibrillar degradation, and loosening of inter-fiber connections. Protein oxidation [24], actin structure changes, and membrane damage further reduce hardness and shear force. These results suggest that repeated freeze–thaw cycles reduce muscle structural support, soften texture, and alter water distribution [25]. Previous studies indicate that texture parameters are significantly correlated with water migration and ice crystal damage, serving as important indicators of internal water structure [28]. In this study, TPA and shear force data provide key physical and structural information for developing MRI-based WHC prediction models, supporting the establishment of accurate and nondestructive predictive models.

### 3.4. MRI T_2_-Weighted Scan Feature Analysis

**Figure 4:**
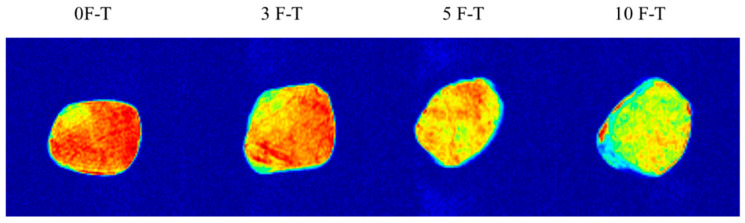
T_2_-weighted pseudo-color images of pork during repeated freeze–thaw cycles.

In T_2_-weighted pseudo-color images, red areas indicate strong proton signals (high water content), while blue areas indicate weak signals (low water content). As shown in Figure 4, fresh pork (0 F–T) exhibited large red areas, indicating uniform water distribution with abundant free and bound water. When increasing the F–T cycles from 1 to 10, red regions gradually decreased, and in 10 F–T samples only a small amount remained at the edges while blue areas expanded. This signal change clearly reflects water loss and redistribution during freeze–thaw. Ice crystal formation and recrystallization damages cell membranes, converting less-mobile water into free water that is lost, which is consistent with moisture loss during repeated freeze–thaw cycles [4,5]. Similar observations have been reported using LF-NMR and MRI, showing reduced water signals and altered spatial distribution during freeze–thaw [29,30]. These results demonstrate that MRI, especially T_2_-weighted imaging, can qualitatively reflect internal water distribution and loss based on signal intensity and spatial patterns.

### 3.5. MRI Scan Selection

**Figure 5:**
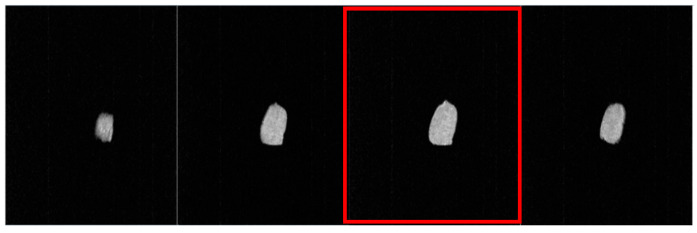
Original image of T2-weighted image.

**Figure 6:**
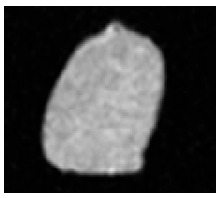
Selected T2-weighted image.

Among multi-slice MRI data, slice completeness and image quality are critical for accurate feature extraction and modeling. Missing edges or cropping may truncate water signal regions, alter pixel intensity distributions, disrupt texture continuity, and introduce bias, reducing feature stability and prediction accuracy. In meat MRI and computer vision studies, poor image integrity impairs reliable classification between fresh and freeze–thaw samples [31]. Four T_2_-weighted slices were acquired per sample; some slices had edge loss due to misplacement (Figure 5). To mitigate potential operator-dependent bias and ensure signal purity, a standardized manual selection was performed by a single experienced researcher to strictly exclude slices containing magnetic susceptibility artifacts or non-muscle tissues. By selecting slices with clear contours and complete coverage (Figure 6), extracted MRI features better represent overall water distribution and correlate more strongly with measured WHC, improving model accuracy and interpretability.

### 3.6. PCR Prediction Model Construction

**Figure 7:**
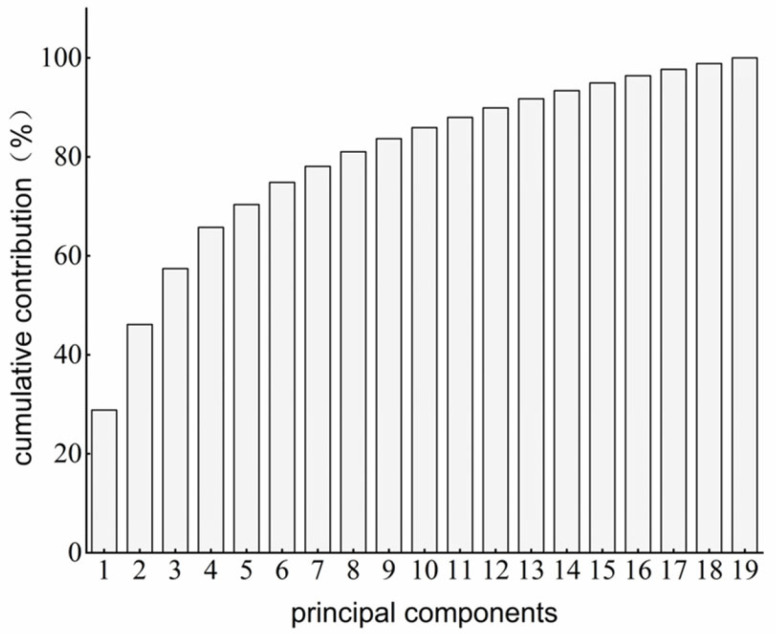
Cumulative contribution rate of principal components in PCR model.

To achieve accurate prediction of pork water-holding capacity (WHC), T_2_-weighted MRI scan features of pork with different WHCs were used as input variables, and pressing loss was used as the output variable. A PCR model was constructed in MATLAB using the PCR function. Principal component analysis (PCA) was applied to the target matrix to identify core components. Sixteen principal components were extracted with a cumulative contribution of 96.39%, of which PC1, PC2, and PC3 contributed 28.82%, 25.61%, and 11.28%, respectively. These results indicate that the first 16 components explain over 95% of the variance, effectively retaining the original information. Using these 16 components as predictors, the PCR model was established (Figure 7). The calibration set had $R_{c}^{2}=0.8825$ and RMSEC = 1.7959, while the validation set had $R_{p}^{2}=0.8856$ and RMSEP = 2.0284. These results indicate that the PCR model can predict pork WHC reasonably well, although the slightly higher RMSEP in the validation set may be due to loss of some WHC-relevant features during principal component extraction [32].

As shown in Table 2, the PCR model achieved good performance, with similar results for the calibration and cross-validation sets. The correlation coefficients ($R_{c}^{2}$ and $R_{p}^{2}$) were both above 0.8, and the RMSEC and RMSEP were 1.7959 and 2.0284, respectively.

### 3.7. PLSR Prediction Model Construction

**Figure 8:**
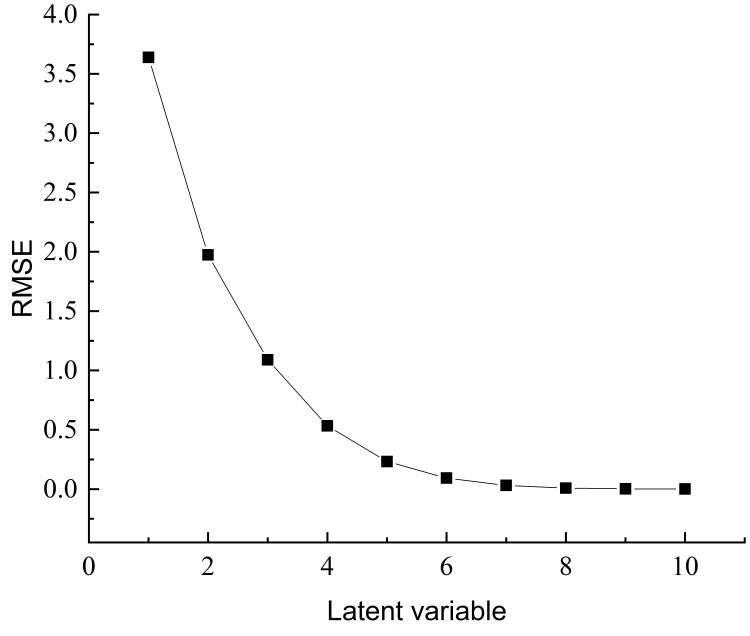
Determination of optimal latent variables in PLSR model.

To compare model performance, the same dataset used for PCR was applied to establish a PLSR model in MATLAB using the pls_nipals function. Monte Carlo cross-validation (MCCV) was used to determine the optimal number of latent variables to improve model fitting. In this study, an 8:2 split was repeated 1000 times, and six latent variables were determined as optimal. Figure 8 shows the relationship between RMSE and latent variable number, with the minimum RMSE at six latent variables. These six variables were used to construct the final PLSR model.

**Figure 9:**
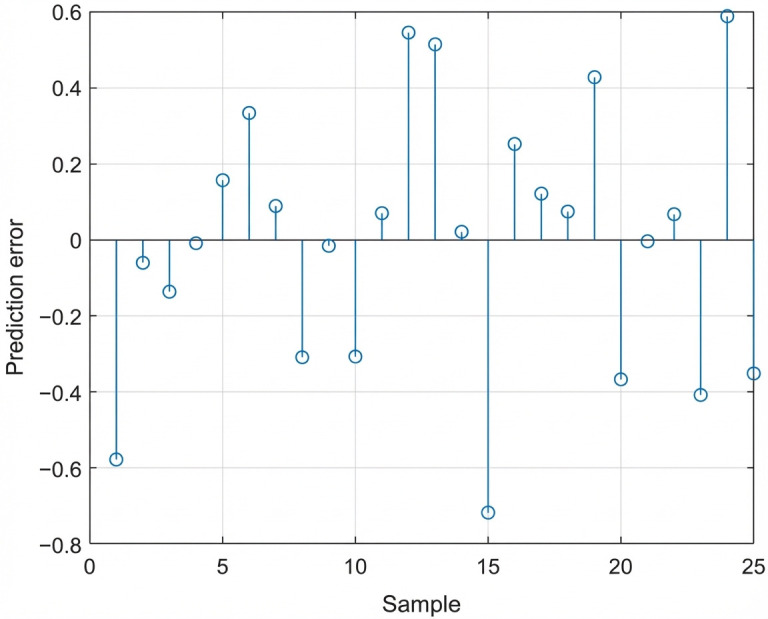
Residual distribution of PLSR model.

As shown in Table 2, the PLSR model achieved $R_{c}^{2}=0.9634$ and RMSEC = 1.0026 for the calibration set and $R_{p}^{2}=0.9656$ and RMSEP = 1.1119 for the validation set, demonstrating significantly better performance than the PCR model. Residual analysis indicated that standard residuals were all less than 1 and evenly distributed without obvious outliers (Figure 9), suggesting high accuracy and stability of the model predictions.

A model is considered highly predictive when $R^{2}>0.90$, good when $0.81\leq R^{2}\leq 0.90$, and moderately predictive when $0.66\leq R^{2}\leq 0.80$. Lower RMSE values indicate better model performance, and residuals below 1 indicate reliability. Both PCR and PLSR are suitable for evaluating pork WHC; however, PLSR shows higher correlation coefficients and lower RMSE values for both calibration and validation sets, indicating superior accuracy and reliability. This demonstrates that PLSR provides better performance for WHC prediction. However, to enhance the model’s generalizability, future work will focus on expanding the biological diversity of the dataset to calibrate the model for application.

## 4. Conclusions

This study systematically investigated the effects of freeze–thaw cycles on pork moisture status, water-holding capacity, and quality characteristics using fresh and repeatedly frozen–thawed pork samples. MRI combined with chemometric methods was applied to develop a nondestructive WHC prediction model. Result showed that with increasing freeze–thaw cycles, pork moisture content significantly decreased, drip loss increased, and overall WHC deteriorated. T_2_-weighted MRI pseudo-color maps visually reflected these changes, with high-proton-signal (red) regions decreasing with increasing cycles, consistent with measured moisture content and WHC trends. Both PCR and PLSR models based on MRI features effectively predicted pork WHC. The PCR model explained 96.394% of the variance using 16 principal components, showing good predictive ability, though some key WHC-related information may be lost during dimensionality reduction. The PLSR model, by considering the covariance between predictors and responses, more fully captured the relationship between MRI features and WHCs, achieving higher correlation coefficients and lower prediction errors for both calibration and validation sets. The PLSR model demonstrated superior stability and generalization compared with PCR. Overall, MRI combined with chemometric methods, particularly PLSR, allows rapid, nondestructive, and accurate prediction of pork WHC, providing a reliable approach for assessing quality changes under repeated freeze–thaw conditions. However, since no sensory analysis or flavor-related indicators were evaluated in this study, the specific implications for consumer acceptability are inferred from physicochemical changes and warrant further verification through sensory panels. This study provides a scientific basis for pork processing, storage, and cold-chain quality monitoring, and lays a foundation for the application of MRI in online and intelligent meat quality evaluation.

## Figures and Tables

**Table 1:** Changes in texture properties of pork.

	0 F–T	3 F–T	5 F–T	10 F–T
Hardness/gf	2836.34 ± 146.04 ^a^	2168.06 ± 122.65 ^b^	1359.97 ± 51.46 ^c^	790.88 ± 49.08 ^d^
springiness/N	0.54 ± 0.020 ^a^	0.45 ± 0.020 ^b^	0.45 ± 0.016 ^b^	0.41 ± 0.024 ^b^
Chewiness/gf	877.49 ± 57.41 ^a^	531.10 ± 42.69 ^b^	297.64 ± 30.09 ^c^	146.93 ± 7.39 ^d^
gumminess/gf	1627.13 ± 106.93 ^a^	1170.73 ± 76.84 ^b^	661.31 ± 49.96 ^c^	366.35 ± 23.01 ^d^
Cohesiveness	0.57 ± 0.016 ^a^	0.54 ± 0.020 ^a^	0.48 ± 0.020 ^b^	0.46 ± 0.012 ^b^
resilience	0.16 ± 0.008 ^a^	0.15 ± 0.008 ^a^	0.13 ± 0.008 ^b^	0.11 ± 0.008 ^b^

Note: Different letters indicate significant differences (*p* < 0.05). Data are expressed as mean ± SEM.

**Table 2:** Relationship between predicted values of PCR and PLSR model with actual pressing loss.

	Calibration Set		Validation Set	
PCR	Rc^2^ = 0.8825	RMSEC = 1.7959	Rp^2^ = 0.8856	RMSEP = 2.0284
PLSR	Rc^2^ = 0.9634	RMSEC = 1.0026	Rp^2^ = 0.9656	RMSEP = 1.1119

## Data Availability

The original contributions presented in the study are included in the article, further inquiries can be directed to the corresponding author.
